# Two new species of *Echinoderes* (Kinorhyncha, Cyclorhagida), *E. romanoi* sp. n. and *E. joyceae* sp. n., from the Gulf of Mexico

**DOI:** 10.3897/zookeys.594.8623

**Published:** 2016-05-30

**Authors:** Stephen C. Landers, Martin V. Sørensen

**Affiliations:** 1 Department of Biological and Environmental Sciences, 210A MSCX, Troy University, Troy AL 36082, USA; 2 Section for GeoGenetics, Natural History Museum of Denmark, University of Copenhagen, Øster Voldgade 5-7, DK-1350 Copenhagen, Denmark

**Keywords:** Echinoderidae, kinorhynchs, meiofauna, morphology, taxonomy

## Abstract

Meiofauna sampling on the continental shelf of the northern Gulf of Mexico has been ongoing since 2007, on annual cruises in collaboration with the National Marine Fisheries Service laboratory in Pascagoula, Mississippi. This sampling has resulted in numerous new species of kinorhynchs from the shelf sediment, two of which are described in detail in this paper. Other species descriptions from this research effort include *Echinoderes augustae*, *Echinoderes skipperae*, and *Echinoderes charlotteae*. We now describe *Echinoderes romanoi* **sp. n.** and *Echinoderes joyceae* **sp. n.**, which are unique in their spine, tube, and glandular cell outlet patterns.

## Introduction

Diversity in the phylum Kinorhyncha has been underreported from the Gulf of Mexico, though recently new investigations are adding to our knowledge of the Gulf species. Currently there are few kinorhynchs identified to species from the Gulf, though their presence and abundance are well documented. The known species diversity in the Gulf of Mexico includes *Echinoderes steineri* (Chitwood, 1951), *Echinoderes coulli* Higgins 1977, *Echinoderes remanei* (Blake, 1930), *Kinorhynchus langi* (Higgins, 1964), Campyloderes cf. vanhoeffeni Zelinka, 1913, *Centroderes barbanigra* Neuhaus et al., 2014, Centroderes cf. drakei Neuhaus et al., 2014, *Echinoderes skipperae* Sørensen & Landers, 2014, *Echinoderes augustae* Sørensen & Landers, 2014, *Echinoderes bookhouti* Higgins, 1964 and *Echinoderes charlotteae* Sørensen et al., 2016 (Chitwood 1951, Harper et al. 1981, Shirley 2009, Neuhaus 2013, Neuhaus et al. 2014, Sørensen and Landers 2014, Fleeger et al. 2015, Sørensen et al. 2016). The last four Gulf records resulted from meiofauna surveys conducted by our labs in the Gulf in collaboration with the National Marine Fisheries Service (NMFS) lab in Pascagoula, Mississippi. This collaboration began in 2007 and continues currently, with sediment collection occurring on annual fall cruises. The first two reports from the kinorhynch analysis of this long term meiofauna study described three new species, *Echinoderes augustae*, *Echinoderes skipperae*, and *Echinoderes charlotteae*, and also provided a redescription of *Echinoderes bookhouti* (Sørensen and Landers 2014, Sørensen et al. 2016). This current contribution is the third in our series of new kinorhynch species discovered in the northern Gulf of Mexico, from continental shelf sediments, and describes *Echinoderes romanoi* sp. n. and *Echinoderes joyceae* sp. n. These new descriptions will be helpful for future taxonomic and morphological studies.

## Materials and methods

**Figure 1.**
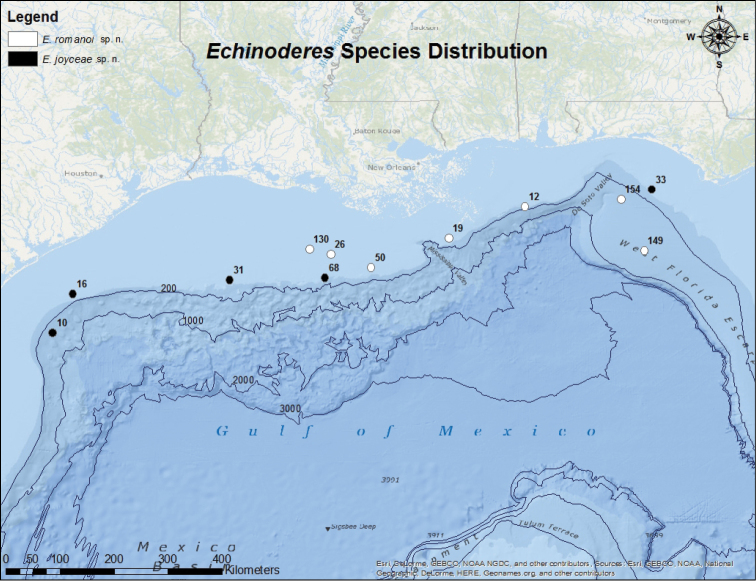
Map of the northern Gulf of Mexico indicating collection stations for *Echinoderes romanoi* sp. n. and *Echinoderes joyceae* sp. n.

Sediment was collected along the northern Gulf of Mexico continental shelf during several NOAA cruises from 2010 to 2015 in collaboration with the NMFS lab in Pascagoula, Mississippi, on NOAA ships *Gordon Gunter*, *Pisces*, and *Oregon II*. Sediment was collected in 2010–2012 using a Shipek® sediment grab, and in 2013–2015 using an Ocean Instruments® mini-multicorer. Specimens from the present study were obtained from 12 locations located along the northern Gulf of Mexico continental shelf (Fig. 1, Table 1).

**Table 1.** Summary of data on stations, species identities and catalogue numbers (KIN-) for specimens deposited at the Natural History Museum of Denmark.

Station	Date	Position	Depth,
Salinity (ppt)	Species	Mounting	Type status and catalogue numbers
149-2010	Nov. 21, 2010	28° 31'9"N	
86° 18'54"W (28.5192°, -86.3151°)	443 m		
35.2	*Echinoderes romanoi* sp. n.	LM	1 ♂ nontype
154-2010	Nov. 22, 2010	29° 22'28"N	
86° 41'46"W (29.3747°, -86.6962°)	382 m		
35.4	*Echinoderes romanoi* sp. n.	LM	1 ♂ paratype, KIN-967, 1 ♂ and 1♀ non-types
050-2013	Nov. 2, 2013	28° 14'21.12"N	
90° 52'1.91"W			
(28.2392°, -90.8672°)	74 m		
36.5	*Echinoderes romanoi* sp. n.	LM	1 ♂ paratype, KIN-899
012-2014	Nov. 13, 2014	29° 15'10"N	
88° 18'23"W (29.2528°, -88.3066°)	90 m		
36.3	*Echinoderes romanoi* sp. n.	SEM	
LM	1 ♂ nontype		
1 ♂ holotype, KIN-962, 2 ♀ paratypes, KIN-963 to KIN-964			
019-2014	Nov. 14, 2014	28° 43'52"N	
89° 34'16"W (28.7313°, -89.5712°)	94 m		
36.3	*Echinoderes romanoi* sp. n.	LM	2 ♀ paratypes, KIN-965 to KIN-966
1 ♂ nontype			
026-2014	Nov. 16, 2014	28° 27 25"N	
91° 31 53"W (28.4572°, -91.5314°)	52 m		
36.2	*Echinoderes romanoi* sp. n.	SEM	
LM	1 ♀ non type		
1 ♀ non type			
130-2015	Nov. 4, 2015	28° 32'45"N	
91° 53'21"W			
(28.5459 °, -91.8894°)	45 m		
36.0	*Echinoderes romanoi* sp. n.	SEM	1 ♀ non type
010-2010	Oct. 16, 2010	27°09'12"N	
96°09'59"W			
(27.1534°,-96.1666°)	427 m		
35.2	*Echinoderes joyceae* sp. n.	LM	1 ♂ holotype KIN-845,
1 ♀ paratype KIN-849			
016-2010	Oct. 17, 2010	27°47'58"N	
95°38'11"^W^			
(27.7995°, -95.8345°)	57 m		
36.4	*Echinoderes joyceae* sp. n	LM	1 ♂ paratype KIN-850
068-2012	Oct. 22, 2012	28°05'12"N	
91°38'59"W			
(28.0868°, -91.6365°)	99 m		
36.4	*Echinoderes joyceae* sp. n	LM	1 ♂ paratype KIN-922
031-2013	Oct. 30, 2013	28°01'55"N	
93°13'29"W			
(28.0320°, -93.2249°)	99 m		
31.2	*Echinoderes joyceae* sp. n	LM	1 ♂ paratype KIN-867
033-2014	Oct. 20, 2014	29° 32'32"N	
86 °11'33"W			
(29.5423°, -86.1926°)	98 m		
35.3	*Echinoderes joyceae* sp. n	SEM	1 ♀ nontype

The samples were fixed immediately in 5–10% formalin on the cruise, and the meiofauna was subsequently extracted by Ludox centrifugation (Burgess 2001). After sorting the animals using a counting wheel, the kinorhynchs were stored in 70% isopropanol. They were processed for light microscopy by subjecting them to increasing concentrations of glycerin before mounting them in Fluoromount G® (Sørensen and Pardos 2008). They were examined and photographed using a Nikon E600 (Troy University) or an Olympus BX51 (University of Copenhagen) light microscope equipped with Nomarski interference contrast optics using digital cameras. Line art illustrations were based on mounted specimens that were drawn using Adobe Illustrator® CS6 or Adobe Photoshop Elements® software. Measurements were made with CellSens® software. All dimensions reported in the tables are based on LM measurements. All light microscopy type material is deposited at the Natural History Museum of Denmark (Copenhagen, Denmark).

Specimens for scanning electron microscopy were observed at the Auburn University Research Instrumentation Facility (Auburn, Alabama). Specimens stored in 70% isopropanol were hydrated, post-fixed in OsO_4_ vapor, then dehydrated to 100% ethanol through a graded series, critical point dried, mounted on aluminum stubs, and sputter-coated with gold. Specimens were photographed with a Zeiss EVO 50 SEM, using the backscatter and secondary electron detectors.

## Results

### Class Cyclorhagida (Zelinka, 1896) Sørensen et al., 2015 Order Echinorhagata Sørensen et al., 2015 Family Echinoderidae Zelinka, 1894 Genus *Echinoderes* Claparède, 1863

#### Echinoderes romanoi sp. n.

Taxon classificationAnimaliaEchinorhagataEchinoderidae

http://zoobank.org/E6387ED6-A68B-4DB2-A835-777A585D5DEC

Figs 2 , 3 , 4

##### Material.

Holotype: Adult male (ZMUC KIN-962), collected from sediment on November 13, 2014, at station 012-2014 (Fig. 1), at 90 m depth, <100 km east of the outlet of the Mississippi River, Louisiana (29°15'10"N, 88°18'23"W), mounted in Fluoromount G®, deposited at the Natural History Museum of Denmark. Paratypes include two females (ZMUC KIN-963 and KIN-964) from station 012-2014, two females (ZMUC KIN-965 and KIN-966) from station 19-2014, one male from station 154-2010 (ZMUC KIN-967) and one male from 050-2013 (ZMUC KIN-899). All paratypes are mounted in Fluoromount G® and deposited at the Natural History Museum of Denmark. Additional nontype material is listed in Table 1. See Figure 1 for localities and Table 1 for detailed station information.

##### Diagnosis.

*Echinoderes* with middorsal spines on segments 4–8, and spines in lateroventral positions on segments 6–9. Tubes present in lateroventral position on segment 5. Glandular cell outlets type 2 present in subdorsal, laterodorsal, sublateral, and ventrolateral positions on segment 2, in midlateral position on segment 5, and in sublateral position on segment 8.

##### Description.

**Figure 2.**
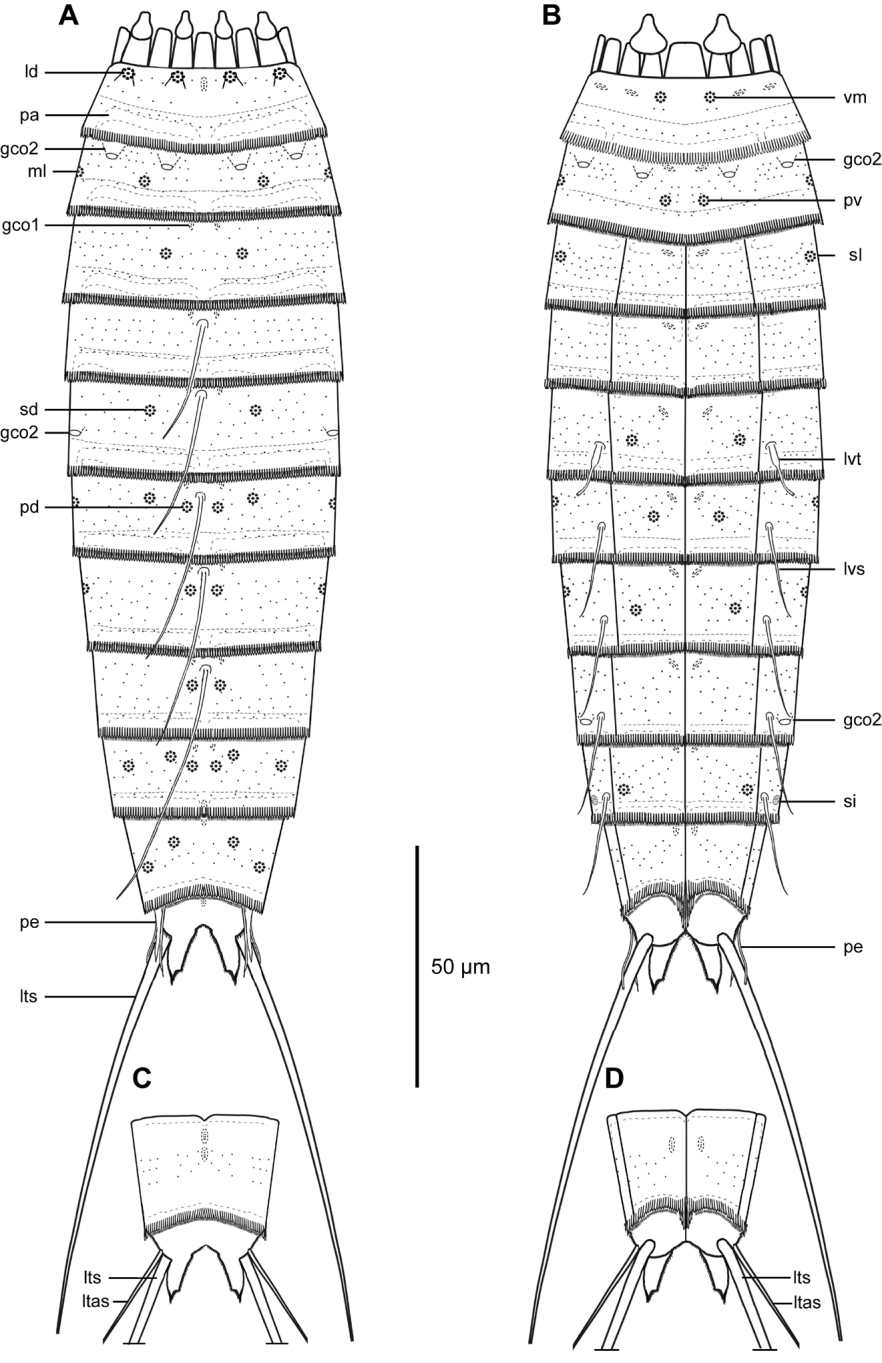
Line art illustrations of *Echinoderes romanoi* sp. n. **A** Male, dorsal view **B** Male, ventral view **C** Female, segments 10 to 11, dorsal view **D** Female, segments 10 to 11, ventral view. Abbreviations: gco1/2, glandular cell outlet type 1/2; ld, laterodorsal sensory spot; ltas, lateral terminal accessory spine; lts, lateral terminal spine; lvs, lateroventral spine; lvt, lateroventral tube; ml, midlateral sensory spot; pa, pachycyclus; pd, paradorsal sensory spot; pe, penile spine; pv, paraventral sensory spot; sd, subdorsal sensory spot; si, sieve plate; sl, sublateral sensory spot; vm, ventromedial sensory spot.

**Figure 3.**
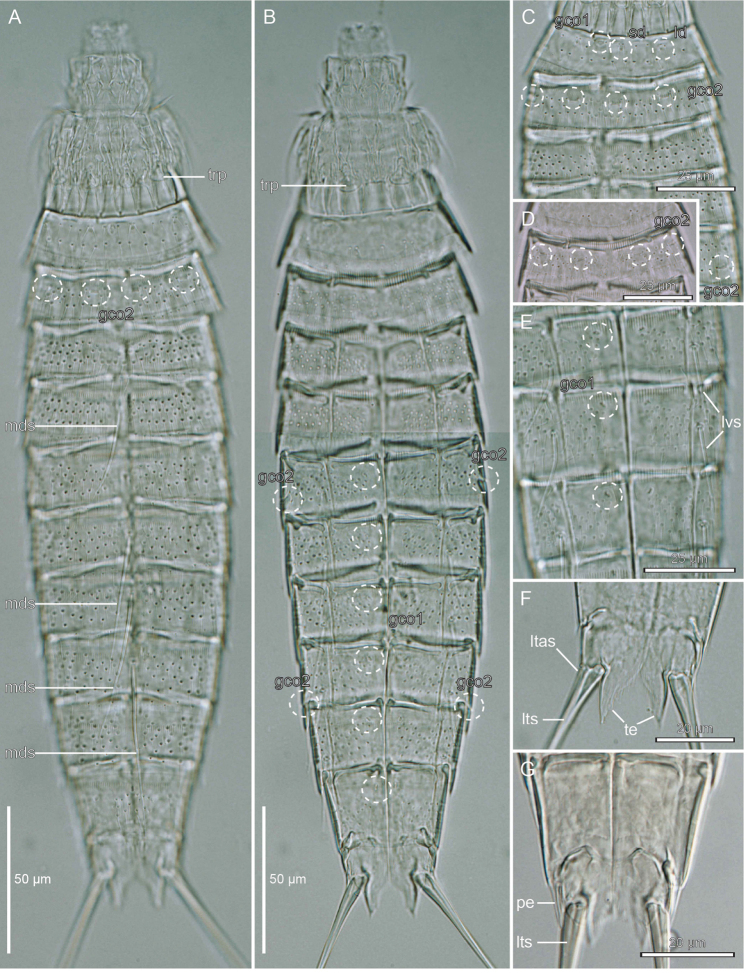
Light micrographs showing overviews and details in male holotype ZMUC KIN-962 (**A–B, D**), female paratypes ZMUC KIN-965 (**C, F**) and ZMUC KIN-966 (**E**) and male paratype ZMUC KIN-967 (**G**) of *Echinoderes romanoi* sp. n. **A.** Dorsal overview **B** Ventral overview **C** Segments 1 to 5, dorsal view **D** Segments 1 and 2, ventral view **E** Segments 6 to 8, ventral view **F** Segments 10 and 11 of a female, ventral view **G** Segments 10 and 11 of a male, ventral view. Abbreviations: gco1/2, glandular cell outlet type 1/2; ld, laterodorsal sensory spot; ltas, lateral terminal accessory spine; lts, lateral terminal spine; lvs, lateroventral spine; mds, middorsal spine; pe, penile spine; sd, subdorsal sensory spot; te, tergal extensions; trp, trichoscalid plate.

**Figure 4.**
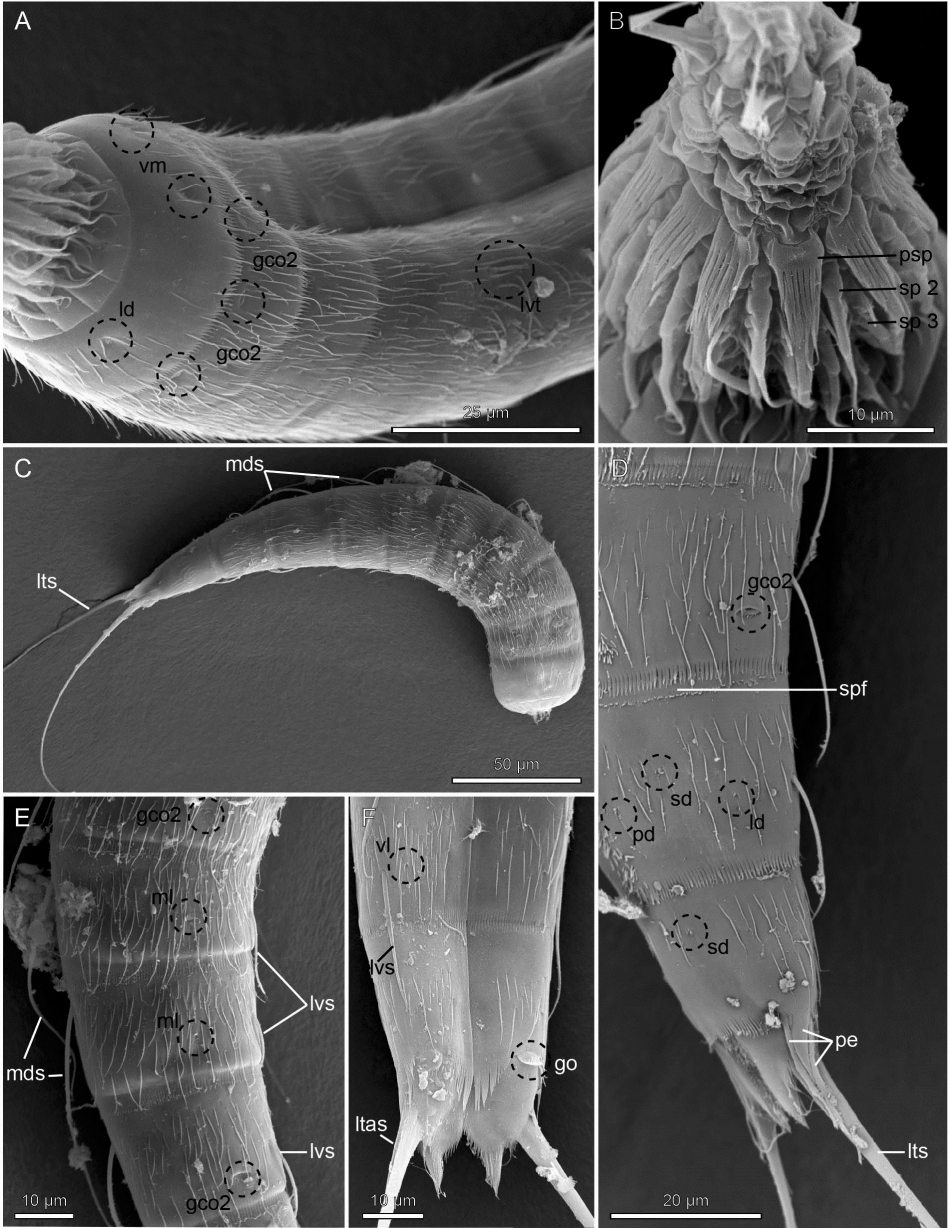
Scanning electron micrographs showing overviews and details in head and trunk morphology of *Echinoderes romanoi* sp. n. **A** Right lateroventral view of anterior end of a female **B** Oral stylets and introvert **C** Right lateral overview of a male **D** Right lateral view of segments 8 to 11 of a male **E** Trunk segments 5 to 8, lateral view **F** Segments 9 to 11 of a female, ventral view. Abbreviations: gco2, glandular cell outlet type 2; go, gonopore; ld, laterodorsal sensory spot; ltas, lateral terminal accessory spine; lts, lateral terminal spine; lvs, lateroventral spine; lvt, lateroventral tube; mds, middorsal spine; ml, midlateral sensory spot; pd, paradorsal sensory spot; pe, penile spine; psp, primary spinoscalid; sd, subdorsal sensory spot; sp2–3, spinoscalids of Rings 2 to 3; spf, secondary pectinate fringe; vl, ventrolateral sensory spot; vm, ventromedial sensory spot.

Adults with head, neck and eleven trunk segments, ranging from 196–247 µm in trunk length (Figs 2–4). For complete overview of measures and dimensions, see Table 2. Distribution of cuticular structures, i.e., sensory spots, glandular cell outlets, spines and tubes, is summarized in Table 3.

**Table 2.** Measurements from light microscopy of *Echinoderes romanoi* sp. n. (in µm) from the Gulf of Mexico, including number of measured specimens (*n*) and standard deviation (SD). Abbreviations: (ac): acicular spine; LTAS: lateral terminal accessory spine; LTS: lateral terminal spine; LV: lateroventral; MD, middorsal; MSW-7: maximum sternal width, measured on segment 7 in this species; S: segment lengths; SW-10, standard width, always measured on segment 10; TL: trunk length.

Character	n	Range	Mean	SD
TL	10	196–247	227	15.08
MSW-7	10	37–44	39	2.28
MSW-7/TL	10	15–20.8%	17.3%	1.87%
SW-10	10	30–35	33	1.58
SW-10/TL	10	13–16.5%	14.4%	1.15%
S1	10	20–24	22	1.33
S2	10	20–24	22	1.45
S3	10	21–26	24	1.51
S4	10	21–27	24	1.75
S5	10	24–29	26	1.64
S6	10	25–30	28	1.51
S7	10	27–32	30	1.57
S8	10	27–33	31	1.77
S9	10	29–34	32	1.58
S10	10	28–33	30	1.83
S11	10	22–30	25	2.42
MD4 (ac)	10	23–49	30	7.23
MD5 (ac)	8	24–55	39	10.04
MD6 (ac)	9	34–65	46	9.30
MD7 (ac)	10	36–68	50	9.54
MD8 (ac)	10	48–73	62	8.15
LV6 (ac)	10	19–35	25	5.57
LV7 (ac)	10	19–36	27	6.00
LV8 (ac)	10	18–42	27	6.81
LV9 (ac)	8	19–46	28	7.87
LTS	10	127–232	154	32.18
LTS/TL	10	55.8–109.4%	68.4%	16.91%
LTAS	5	45–77	62	13.38

**Table 3.** Summary of nature and location of sensory spots, glandular cell outlets, tubes and spines arranged by series in *Echinoderes romanoi* sp. n. Abbreviations: LA: lateral accessory; LD: Laterodorsal; LV: lateroventral; MD: middorsal; ML: midlateral; PD: paradorsal; PV: paraventral; SD: subdorsal; SL: sublateral; VL: ventrolateral; VM: ventromedial; ac, acicular spine; gco 1/2, glandular cell outlet type 1/2; ltas, lateral terminal accessory spine; lts, lateral terminal spine; pe, penile spines; si, sieve plate; ss, sensory spot; tu, tube; (♀), female and (♂), male conditions of sexually dimorphic characters.

Position Segment	MD	PD	SD	LD	ML	SL	LA	LV	VL	VM	PV
1	gco1		ss	ss		gco1		gco1		ss	
2			gco2	gco2, ss	ss	gco2			gco2		gco1, ss
3		gco1	ss			ss					gco1
4	ac	gco1									gco1
5	ac	gco1	ss		gco2			tu		ss	gco1
6	ac	gco1, ss	ss		ss			ac		ss	gco1
7	ac	gco1 ,ss			ss			ac		ss	gco1
8	ac	gco1, ss				gco2		ac			gco1
9		gco1, ss	ss	ss		si		ac	ss		gco1
10	gco1, gco1		ss	ss							gco1
11	gco1, gco1				pe(♂)		ltas(♀)	lts			

The head (Fig. 3A, B, 4B) consists of a retractable mouth cone and an introvert. The mouth cone has nine outer oral styles. The introvert sectors are defined by 10 primary spinoscalids in ring 1. Each primary spinoscalid consists of a basal sheath with approximately 7 long extensions forming a fringed margin, and a distal end piece with a blunt tip. It was only possible to obtain information about the appearance and arrangement of scalids for introvert sectors 2, 3, and 4, which have the following characteristics: single central scalids of Rings 02 and 04, and paired scalids of Rings 03 and 05.

The neck (Figs 2A, B, 3A, B) has 16 placids, measuring 10 µm in length. The midventral placid is broadest, measuring 9 µm in width at its base, whereas all other are narrower, measuring 5–6 µm in width at their bases. The trichoscalid plates, each with a trichoscalid, are present in subdorsal, laterodorsal and ventromedial positions.

Segment 1 (Figs 2A, B, 3A–C, 4A) consists of a complete cuticular ring. Sensory spots are located anteriorly in subdorsal, laterodorsal, and ventromedial positions; sensory spots minute and rounded with two anterior cuticular hairs. Glandular cell outlets type 1 present middorsally, sublaterally and lateroventrally. Cuticular hairs sparse on dorsal and ventral surface. A line of cuticular hairs is located below the intersegmental joint line. Pectinate fringe of posterior segment margin with typical fringe tips.

Segment 2 (Figs 2A, B, 3A–D, 4A) consists of a complete cuticular ring. Pachycyclus of the anterior segment margin interrupted in middorsal and lateroventral positions. Sensory spots located in laterodorsal, midlateral, and paraventral positions; sensory spots on this and following segments minute and rounded. Glandular cell outlets type 2 located in subdorsal, laterodorsal, sublateral and ventrolateral positions. Glandular cell outlets type 1 located in paraventral positions. Secondary pectinate fringe present on this segment and on the following segments 3–10.

Segment 3 (Figs 2A, B, 3A–C), and remaining segments, consisting of one tergal and two sternal plates. Pachycyclus of the anterior segment margin interrupted middorsally, midventrally, and at the tergosternal junctions. Sensory spots present in subdorsal and sublateral positions. Cuticular hairs evenly distributed over tergal and sternal plates, between the pectinate fringe and intersegmental joint line, with a line of cuticular hairs below the joint line. Glandular cell outlets type 1 in paradorsal and paraventral positions.

Segment 4 (Figs 2A,B, 3A, B, 4A) with acicular spine in middorsal position. Sensory spots not present. Glandular cell outlets type 1 slightly anterior to the spine insertion paradorsally and also present in paraventral positions. Pachycycli and cuticular hairs as on preceding segment.

Segment 5 (Figs 2A, B, 3A,B, 4A,C,E) with acicular spine in middorsal position and tubes in lateroventral positions. Sensory spots present in subdorsal and ventromedial positions. Glandular cell outlets type 2 in midlateral position. Pachycycli, glandular cell outlets type 1 and cuticular hairs as on preceding segment.

Segment 6 (Figs 2A, B, 3A, B, E, 4E) with middorsal and lateroventral acicular spines. Sensory spots present in paradorsal, subdorsal, midlateral, and ventromedial positions; ventromedial sensory spots slightly closer to midsternal junction than those on preceding segment. Pachycycli, glandular cell outlets type 1, and cuticular hairs as on preceding segment.

Segment 7 (Figs 2A, B, 3A, B, E, 4C,E) with middorsal and lateroventral acicular spines. Sensory spots present in paradorsal, midlateral, and ventromedial positions; ventromedial sensory spots aligned with those on segment 5. Pachycycli, glandular cell outlets type 1, and cuticular hairs as on preceding segment.

Segment 8 (Figs 2A, B, 3A, B, E, 4D, E) with middorsal and lateroventral acicular spines. Sensory spots present in paradorsal position. Glandular cell outlets type 2 in sublateral position. Pachycycli, glandular cell outlets type 1, and cuticular hairs as on preceding segment.

Segment 9 (Figs 2A, B, 3A, B, 4D,F) with acicular spines in lateroventral position. Sensory spots present in paradorsal, subdorsal, laterodorsal and ventrolateral positions. Glandular cell outlets type 1 are present in paradorsal and paraventral positions. Minute sieve plates present in sublateral position. Cuticular hairs and pachycycli as on preceding segment.

Segment 10 (Figs 2A–D, 3A, B, F, G, 4D, F) with sensory spots in subdorsal and laterodorsal positions. Glandular cell outlets type 1 in tandem at the middorsal position, and in paraventral position. Posterior margin of pectinate fringe curved slightly anteriorly at the middorsal location. Posterior margins of sternal plates slightly rounded. Cuticular hairs sparse.

Segment 11 (Figs 2A–D, 3A, B, F, G, 4D, F) with lateral terminal spines. Sensory spots not observed. Females with thin lateral terminal accessory spines. Female gonopores near anterolateral margins of sternal plates of segment 11; gonopores with rounded, intracuticular thickenings, and externally covered by fringed flap. Males with three pairs of penile spines. The dorsal- and ventral-most penile spines are thin and flexible; medial ones are more stout and rigid, tapering towards the tip. Glandular cell outlets type 1 present in tandem at the middorsal position, with the anterior outlet positioned horizontally and the posterior outlet positioned vertically. Tergal extensions elongated and curved on the lateral surface, with margin of medial sides decorated with hair-like extensions. Sternal extensions are rounded.

##### Etymology.

This species is named after the late Dr. Frank A. Romano III, Jacksonville State University, Alabama, for his contributions to the study of meiofauna and for his initiation of our ongoing meiofauna survey.

##### Remarks.

*Echinoderes romanoi* sp. n. is characterized by the presence of middorsal spines on segments 4 to 8, lateroventral tubes on segment 5, lateroventral spines on segments 6 to 9, and glandular cell outlets type 2 on segments 2 (4 pairs), 5, and 8. This combination of spines, tubes, and glandular cell outlets is unique among all species in the genus. The spine/tube arrangement is not unusual among congeners: 36 additional species share the presence of middorsal spines on segments 4 to 8 and lateroventral tubes/spines on segments 5 to 9, and out of these, ten also lack tubes on segment 2 as does *Echinoderes romanoi* sp. n.: *Echinoderes angustus* Higgins and Kristensen 1988, *Echinoderes aquilonius* Higgins and Kristensen 1988, *Echinoderes tubilak* Higgins and Kristensen 1988, *Echinoderes remanei* (Blake 1930), *Echinoderes brevicaudatus* Higgins 1977, *Echinoderes cernunnos* Sørensen et al. 2012, *Echinoderes koreanus* Adrianov, 1999, *Echinoderes stockmani* Adrianov, 1999, *Echinoderes obtuspinosus* Sørensen et al. 2012, and *Echinoderes bookhouti* Higgins, 1964 (Blake 1930, Higgins 1964a, 1964b, Higgins 1977, Higgins and Kristensen 1988, Adrianov and Malakhov 1999, Sørensen et al. 2012, 2016). Many of the descriptions of these ten species do not include glandular cell outlet type 2 information, though there are a variety of characteristics that distinguish *Echinoderes romanoi* n. sp. from each of the 10 other taxa. The first three species, *Echinoderes angustus*, *Echinoderes aquilonius*, and *Echinoderes tubilak*, all described from Disko Island, Greenland, can be distinguished from *Echinoderes romanoi* n. sp. by size alone. The three Greenland species all have a trunk length and placid length much larger than the trunk length (196–247 µm) and placid length (10 µm) of *Echinoderes romanoi* n. sp. (*Echinoderes angustus* 320–475 µm, 16–20 µm; *Echinoderes aquilonius* 363–465 µm, 15–20 µm; *Echinoderes tubilak* 333–415 µm, 14–18 µm). The same distinction is true for *Echinoderes remanei*, redescribed by Higgins (1964a), which has a trunk length of 282–358 µm. *Echinoderes brevicaudatus* has lateral dorsal tubes on segment 10, and short stubby lateral terminal spines, distinct from the new species. *Echinoderes cernunnos* has glandular cell outlets type 2 located similarly to *Echinoderes romanoi* on segments 2, 5 and 8, though *Echinoderes cernunnos* also has glandular cell outlets type 2 in the midlateral position on segment 7 and additionally has elongated spinous tergal extensions. *Echinoderes koreanus* has spines in the lateral dorsal positions on segments 7 and 8, and tubes in the laterodorsal position on segment 10, unlike the new species. *Echinoderes stockmani* is distinguished by having the lateral spines on segment 8 distinctly longer than those on segment 9, unlike *Echinoderes romanoi* n. sp. *Echinoderes obtuspinosus* has glandular cell outlets type 2 similarly to *Echinoderes romanoi* on segments 2 and 8. However, *Echinoderes obtuspinosus* has glandular cell outlets type 2 in the subdorsal position on segment 4 and none on segment 5. Further, *Echinoderes obtuspinosus* has short stubby lateral terminal spines. Finally, *Echinoderes bookhouti* has lateral accessory spines on segment 8, and lacks glandular cell outlets type two on segment 5 and in the laterodorsal and sublateral position on segment 2.

#### Echinoderes joyceae sp. n.

Taxon classificationAnimaliaEchinorhagataEchinoderidae

http://zoobank.org/B7ED6634-BA3F-4250-84DC-DE7CBC53A19D

Figs 5 , 6 , 7

##### Material.

Holotype: Adult male (ZMUC KIN-845), collected from muddy sediment on October 16, 2010, at station 010-2010 (Fig. 1), at 427 m depth, about 100 km east southeast of Corpus Christi, Texas (27°09'12"N 96°09'59"W), mounted in Fluoromount G®, deposited at the Natural History Museum of Denmark. Paratypes include one female (ZMUC KIN-849), collected at same time and locality as the holotype, and three males, collected at stations 016-2010 (ZMUC KIN-850), 068-2012 (ZMUC KIN-922), and 031-2013 (ZMUC KIN-867). All paratypes were mounted in Fluoromount G® and deposited at the Natural History Museum of Denmark. Additional non-type material includes one female from station 033-2014. See Fig. 1 for localities and Table 1 for detailed station data.

##### Diagnosis.

Conspicuously small *Echinoderes* (183–209 µm) with middorsal spines on segments 4, 6 and 8, and spines in lateroventral positions on segments 6 to 9. Tubes present in ventrolateral positions on segment 2, in lateroventral positions on segment 5, and in laterodorsal positions near the posterior margin of segment 10. Glandular cell outlets type 2 present in subdorsal position on segment 2, in midlateral position on segment 6, and in sublateral position on segment 8.

##### Description.

**Figure 5.**
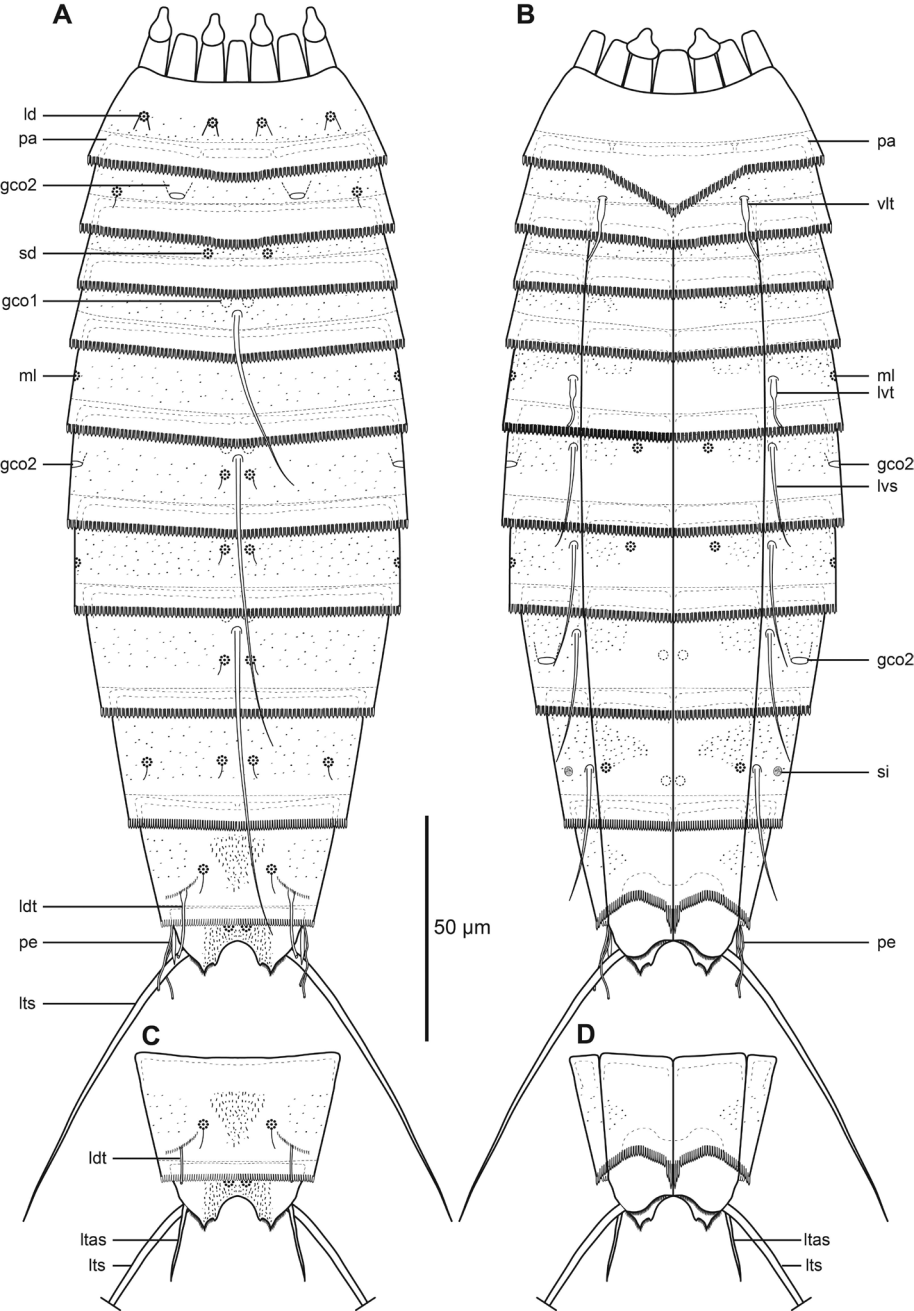
Line art illustrations of *Echinoderes joyceae* sp. n. **A** Male, dorsal view **B** Male, ventral view **C** Female, segments 10 to 11, dorsal view **D** Female, segments 10 to 11, ventral view. Abbreviations: gco1/2, glandular cell outlet type 1/2; ld, laterodorsal sensory spot; ldt, laterodorsal tube; ltas, lateral terminal accessory spine; lts, lateral terminal spine; lvs, lateroventral spine; lvt, lateroventral tube; ml, midlateral sensory spot; pa, pachycyclus; pe, penile spine; sd, subdorsal sensory spot; si, sieve plate; vlt, ventrolateral tube.

**Figure 6.**
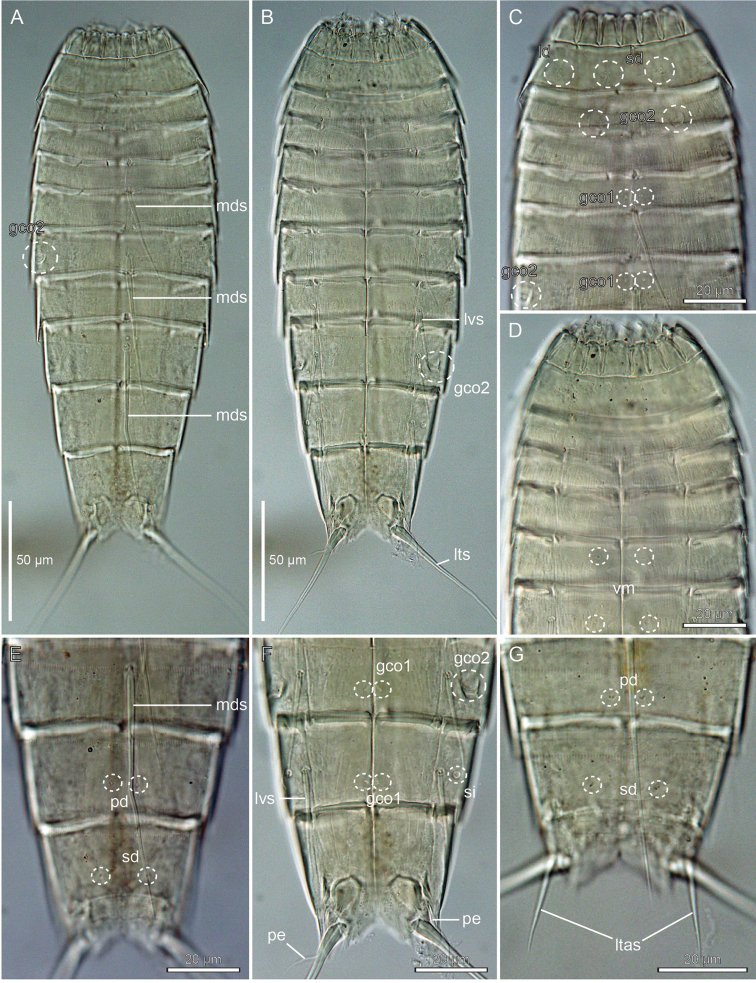
Light micrographs showing overviews and details in male holotype ZMUC KIN-845 (**A–F**), and female paratype ZMUC KIN-849 (**G**) of *Echinoderes joyceae* sp. n. **A** Dorsal overview **B** Ventral overview **C** Segments 1 to 6, dorsal view **D** Segments 1 to 6, ventral view **E** Segments 8 to 11, dorsal view **F** Segments 8 to 11, ventral view **G** Segments 9 to 11, dorsal view. Abbreviations: gco1/2, glandular cell outlet type 1/2; ld, laterodorsal sensory spot; ltas, lateral terminal accessory spine; lts, lateral terminal spine; lvs, lateroventral spine; mds, middorsal spine; pe, penile spine; pd, paradorsal sensory spot; sd, subdorsal sensory spot; si, sieve plate; vm, ventromedial sensory spot.

**Figure 7.**
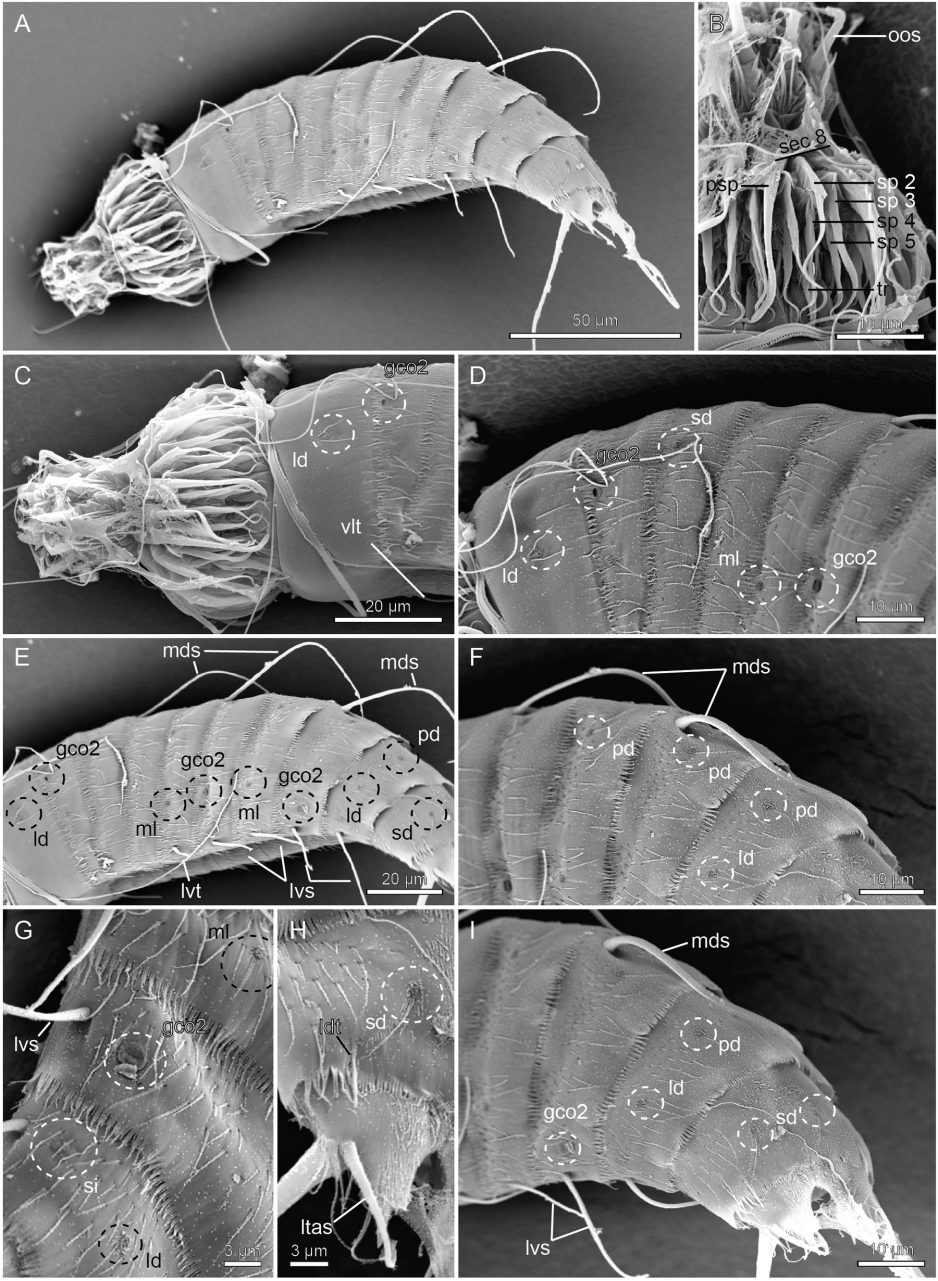
Scanning electron micrographs showing overviews and details in head and trunk morphology of female *Echinoderes joyceae* sp. n. **A** Lateral overview **B** Mouth cone and introvert sector 8 **C** Head and segments 1 to 2, lateral view **D** Laterodorsal parts of segments 1 to 6 **E** Trunk segments 1 to 10, lateral view **F** Laterodorsal parts of segments 4 to 7 **G** Detail showing sublateral parts of segments 7 to 9 **H** Detail showing sub- and laterodorsal parts of segment 10 and left tergal extension of segment 11 **I** Segments 7 to 11, laterodorsal and caudal view. Abbreviations: gco2, glandular cell outlet type 2; ld, laterodorsal sensory spot; ldt, laterodorsal tube; ltas, lateral terminal accessory spine; lvs, lateroventral spine; lvt, lateroventral tube; mds, middorsal spine; ml, midlateral sensory spot; oos, outer oral style; pd, paradorsal sensory spot; psp, primary spinoscalid; sd, subdorsal sensory spot; sec 8, introvert sector 8; si, sieve plate; sp2–5, spinoscalids of rings 2 to 5; tr, trichoscalid; vlt, ventrolateral tube.

Adults conspicuously small (183–209 µm in trunk length), with head, neck and eleven trunk segments (Figs 5–7). For complete overview of measures and dimensions, see Table 4. Distribution of cuticular structures, i.e., sensory spots, glandular cell outlets, spines and tubes, is summarized in Table 5.

**Table 4.** Measurements from light microscopy of *Echinoderes joyceae* sp. n. (in µm) from the Gulf of Mexico, including number of measured specimens (*n*) and standard deviation (SD). Abbreviations: (ac): acicular spine; LTAS: lateral terminal accessory spine; LTS: lateral terminal spine; LV: lateroventral; MD, middorsal; MSW-6: maximum sternal width, measured on segment 6 in this species; S: segment lengths; SW-10, standard width, always measured on segment 10; TL: trunk length.

Character	*n*	Range	Mean	SD
TL	4	183–209	195	10.90
MSW-6	4	40–46	43	3.00
MSW-6/TL	3	20.4–23.0%	21.8%	1.32%
SW-10	4	34–37	35	1.26
SW-10/TL	3	16.7–19.4%	17.8%	1.38%
S1	4	23–26	25	1.50
S2	4	22–26	25	1.73
S3	4	23–25	23	1.26
S4	4	21–26	24	2.22
S5	4	23–27	26	1.73
S6	4	25–30	28	2.22
S7	4	27–31	30	1.91
S8	4	30–33	32	1.29
S9	4	30–31	31	0.58
S10	4	31–33	32	0.82
S11	4	24–27	25	1.41
MD4 (ac)	5	40–58	47	7.30
MD6 (ac)	5	59–74	66	6.07
MD8 (ac)	5	61–75	68	7.06
LV6 (ac)	4	20–25	23	2.06
LV7 (ac)	4	22–31	27	3.70
LV8 (ac)	4	29–39	32	5.12
LV9 (ac)	4	31–34	33	1.29
LTS	5	72–83	79	4.66
LTS/TL	4	39.3–42.9%	40.3%	1.75%
LTAS	1	24	N/A	N/A

**Table 5.** Summary of nature and location of sensory spots, glandular cell outlets, tubes and spines arranged by series in *Echinoderes joyceae* sp. n. Abbreviations: LA: lateral accessory; LD: Laterodorsal; LV: lateroventral; MD: middorsal; ML: midlateral; PD: paradorsal; PV: paraventral; SD: subdorsal; SL: sublateral; VL: ventrolateral; VM: ventromedial; ac, acicular spine; gco 1/2, glandular cell outlet type 1/2; ltas, lateral terminal accessory spine; lts, lateral terminal spine; pe, penile spines; si, sieve plate; ss, sensory spot; tu, tube; (♀), female and (♂), male conditions of sexually dimorphic characters.

Position Segment	MD	PD	SD	LD	ML	SL	LA	LV	VL	VM	PV
1			ss	ss							
2			gco2	ss					tu		
3			ss								
4	ac	gco1									
5					ss			tu			
6	ac	gco1,ss			gco2			ac		ss	
7		ss			ss			ac		ss	
8	ac	gco1,ss				gco2		ac			gco1
9		ss		ss		si		ac	ss		gco1
10			ss	tu							
11		ss			pe(♂)		ltas(♀)	lts			

The head (Fig. 7A–C) consists of a retractable mouth cone and an introvert. Mouth cone with nine outer oral styles that alternate in length between slightly shorter and slightly longer ones. No outer oral styles present anterior to introvert sector 6. A fringe with three long spikes is located at the base of each outer oral style. It was only possible to obtain complete information about appearance and arrangement of scalids for introvert sectors 8 and 9. Sector 8: single central scalids of Rings 02 and 04, and paired scalds of Rings 03 and 05. No scalids present posterior to Ring 05, except for a single trichoscalid that attaches through a trichoscalid plate. Sector 9: single central scalids of Rings 02, 04, and 06, and paired scalds of Rings 03 and 05. No trichoscalids present.

The neck (Figs 5A, B, 6A–D) has 16 placids, measuring 11 µm in length. The midventral placid is broadest, measuring 9 µm in width at its base, whereas all other are narrower, measuring 6 µm in width at their bases. The trichoscalid plates, each with a trichoscalid, present in subdorsal, laterodorsal and ventromedial positions.

Segment 1 (Figs 5A, B, 6A–D, 7A, C, D) consists of a complete cuticular ring. Sensory spots are located in subdorsal and laterodorsal positions; sensory spots are rounded and flanked by a pair of cuticular hairs. Glandular cell outlets type 1 not present. Cuticular hairs are very scarce on the dorsal side, and not present at all on the ventral. The posterior segment margin is straight along the dorsal and lateral side, but extends more posteriorly ventrally, into a midventral point. Pectinate fringe of posterior segment margin with fringe tips alternating with small trichoid extensions.

Segment 2 (Fig. 5A, B, 6A–D, 7A, C, D) consists of a complete cuticular ring, with tubes located in ventrolateral position. Sensory spots (with one marginal hair) located in the laterodorsal position. Pachycyclus of the anterior segment margin of regular thickness and interrupted in subdorsal and ventrolateral positions. Glandular cell outlets type 2 are located in subdorsal position. Secondary pectinate fringe not detected on this or any of the following segments. Bracteate cuticular hairs evenly distributed in a medial band around the segment. The posterior segment margin is straight and consists of a pectinate fringe, with fringe tips alternating with small trichoid extensions.

Segment 3 (Figs 5A, B, 6A–D, 7A, D, E), and remaining segments, consisting of one tergal and two sternal plates. Pachycyclus of the anterior segment margin interrupted middorsally; thickness on the dorsal side rather average; ventral pachycycli thicker, and interrupted at the tergosternal and midsternal junctions. Sensory spots (without marginal hairs) are located in subdorsal position only. Cuticular hairs evenly distributed over tergal plate, whereas the sternal plates only have a few hairs near their anterolateral corners. Posterior segment margin and pectinate fringe as on preceding segment.

Segment 4 (Figs 5A, B, 6A–D, 7A, D, E) with acicular spine in middorsal position, flanked by pair of paradorsal glandular cell outlets type 1. Sensory spots not present. Pachycycli, pectinate fringe of posterior margin and cuticular hairs as on preceding segment.

Segment 5 (Figs 5A, B, 6A–D, 7A, D, E) with tubes in lateroventral position. Sensory spots (without marginal hairs) present in midlateral positions. Pachycycli, pectinate fringe of posterior margin and cuticular hairs as on preceding segment.

Segment 6 (Figs 5A, B, 6A–D, 7A, D–F) with middorsal and lateroventral acicular spines. Paradorsal glandular cell outlets type 1 present anterior to middorsal spine, and paradorsal sensory spots (with or without marginal hairs) posterior to spine. One additional pair of sensory spots without marginal hairs present in ventromedial position. Glandular cell outlets type 2 present in midlateral position. Pachycycli, pectinate fringe of posterior margin and cuticular hairs as on preceding segment.

Segment 7 (Figs 5A, B, 6A, B, 7A, E–G, I) with acicular spines in lateroventral position, and sensory spots in paradorsal (with one marginal hair), midlateral and ventromedial positions. Pachycycli, pectinate fringe of posterior margin and cuticular hairs as on preceding segment.

Segment 8 (Figs 5A, B, 6A, B, E, F, 7A, E–G, I) with middorsal and lateroventral acicular spines. Paradorsal glandular cell outlets type 1 present anterior to middorsal spine, and paradorsal sensory spots (with marginal hairs) posterior to spine. Additional glandular cell outlets type 1 are present in paraventral position, and glandular cell outlets type 2 in sublateral position. Pachycycli, pectinate fringe of posterior margin and cuticular hairs as on preceding segment.

Segment 9 (Figs 5A, B, 6 A, B, E–G, 7H, I) with acicular spines in lateroventral position. Sensory spots with marginal hairs present in paradorsal and laterodorsal positions, and without marginal hairs in ventrolateral positions. Glandular cell outlets type 1 are present in paraventral position, and a pair of very minute sieve plates is located in sublateral position. Ventral pachycycli of anterior segment margin slightly thinner than those on preceding segment. Pectinate fringe of posterior margin only with regular fringe tips. Cuticular hairs as on preceding segment on tergal plate; sternal plates with cuticular hairs forming triangular patterns extending from the tergosternal junctions.

Segment 10 (Figs 5A–D, 6A–B, E–G, 7H, I) with laterodorsal tubes near posterior segment margin: tubes in males are well-developed, resembling regular tubes with thickened bases; tubes in females are about half as long and formed like simple tubes without thickened bases; tubes in both sexes emerge through slit-like openings in the cuticle at the posterior part of the segment. Sensory spots with marginal hair present in subdorsal positions. Glandular cell outlets type 1 not observed. Tergal plate with triangular middorsal patch of cuticular hair-like extensions, without perforation sites. Cuticular hairs with perforation sites in two patches going from the laterodorsal positions to the tergosternal junctions; hairs on the sternal plates only ventrolaterally, near the tergosternal junctions. Posterior margin of tergal plate straight; posterior margins of sternal plates concave.

Segment 11 (Fig. 5A–D, 6A–B, E–G, 7H, I) with lateral terminal spines. Females with thin lateral terminal accessory spines, and males with three pairs of penile spines: dorsal- and ventral-most penile spines are thin and flexible; medial ones are more stout and rigid. Sensory spots, without marginal hairs, present in paradorsal positions. Glandular cell outlets type 1 not observed. Cuticular hairs with perforation sites not present. Cuticular hair-like extensions present in patch going from the subdorsal to the middorsal areas. Tergal extensions short and pointed, with margin of inferior sides interrupted by elongated tips formed by the marginal fringes. Sternal extensions are short and broadly rounded.

##### Etymology.

This species is named after Joyce Wright Landers—the wife of the first author.

##### Remarks.

*Echinoderes joyceae* sp. n. is characterized by the presence of middorsal spines on segments 4, 6 and 8, ventrolateral tubes on segment 2, lateroventral tubes/spines on segments 5 to 9, and laterodorsal tubes on segment 10. This combination of spines and tubes is not unusual among congeners, and is shared with five other species: *Echinoderes bermudensis* Higgins 1982, *Echinoderes kristenseni* Higgins 1985 *Echinoderes abbreviatus* Higgins 1983 *Echinoderes hispanicus* Pardos et al., 1998 and *Echinoderes intermedius* Sørensen, 2006. (Higgins 1982, 1983, 1985; Pardos et al. 1998, Sørensen 2006). These species are distinguished from *Echinoderes joyceae* using a number of characteristics. All five of these species have tergal extensions on segment 11 distinct from *Echinoderes joyceae*. Additionally, the new species has a distinctive distribution of glandular cell outlets type 2, with locations in the subdorsal position on segment 2. Amongst the abovementioned species this is only found in *Echinoderes kristenseni*, which also has glandular cell outlets type 2 in lateroventral positions of segment 2, not present in *Echinoderes joyceae* sp. n. The most unique character combination in *Echinoderes joyceae* sp. n. though, is the presence of glandular cell outlets type 2 in midlateral positions of segment 6 and in sublateral positions of segment 8. This combination is not found in any other species of *Echinoderes*. Furthermore, *Echinoderes joyceae* sp. n. is characteristic by its minute size. With a trunk length ranging from 183 to 209 µm, *Echinoderes joyceae* sp. n. is among the smallest known *Echinoderes*.

## Discussion

This study describes *Echinoderes romanoi* sp. n. and *Echinoderes joyceae* sp. n., and reports the known distribution of the two new species along the northern Gulf of Mexico continental shelf. In common with the previous shelf species reported so far during this long term meiofauna sampling is the broad distribution of the taxa. *Echinoderes romanoi* sp. n. and *Echinoderes joyceae* sp. n. are distributed across wide regions of the United States’ Gulf shelf, with the location of *Echinoderes romanoi* extending from central Louisiana to western Florida, and with *Echinoderes joyceae* sp. n. extending from Texas to Florida. Similarly, *Echinoderes augustae*, *Echinoderes skipperae*, *Echinoderes charlotteae*, and *Echinoderes bookhouti* (Sørensen and Landers 2014, Sørensen et al. 2016), all reported during this survey from the northern Gulf shelf, have broad distributions either extending across half of the U.S. Gulf shelf or across the entire Gulf. As more samples are processed during our survey, more locations for all of these species will be determined. Their distribution will likely cover the entire shelf from Florida to Mexico, given the trend observed so far. Despite the broad distribution of these *Echinoderes* species across the Gulf, it is interesting that they have not been observed in coastal marshes. In a recent study on the effects of the Deepwater Horizon oil spill from 2010 in Barataria Bay, Louisiana (Fleeger et al. 2015), all identified kinorhynchs from a subsample of 100 animals were identified as *Echinoderes coulli*, with no offshore species present (identifications in the Fleeger et al. 2015 paper were made by M.V. Sørensen). *Echinoderes coulli* is an estuarine species, which has not been observed in our offshore samples. Our sampling on the continental shelf has consistently sampled sediment from high salinity waters, yielding species that may not tolerate estuarine conditions. Sampling along the U.S. shoreline in high and low salinity waters are needed to determine if the shelf species are found in the intertidal zone or marshes.

## Supplementary Material

XML Treatment for Echinoderes romanoi

XML Treatment for Echinoderes joyceae
